# Minority Dissent and Social Acceptance in Collaborative Learning Groups

**DOI:** 10.3389/fpsyg.2017.00458

**Published:** 2017-03-28

**Authors:** Petru L. Curşeu, Sandra G. L. Schruijer, Oana C. Fodor

**Affiliations:** ^1^Department of Psychology, “Babes-Bolyai” UniversityCluj-Napoca, Romania; ^2^Department of Organisation, Open University of the NetherlandsHeerlen, Netherlands; ^3^Utrecht School of Governance, Utrecht UniversityUtrecht, Netherlands; ^4^Tias School for Business and Society, Tilburg UniversityTilburg, Netherlands

**Keywords:** group cognitive complexity, collaborative learning, minority dissent, social acceptance

## Abstract

The main aim of this paper is to test the extent to which social acceptance moderates the impact of minority dissent on group cognitive complexity (GCC). We hypothesize that divergent views expressed by a minority increase GCC especially when the group climate is open to divergent contributions (e.g., a socially accepting group climate). We also hypothesize that group size has a non-linear association with GCC in such a way that GCC increases as group size increases from low to average and then GCC decreases as group size further increases from average to high. We test these hypotheses in a sample of 537 students (258 women, with an average age of 23.35) organized in 92 groups that have worked together in the same group throughout the semester, and show that: (1) group size has a decreasing positive association with GCC, (2) both minority dissent and social acceptance are beneficial for GCC and (3) groups with the highest GCC are those that experience minority dissent and develop a socially accepting climate (in which group members can equally participate to the task), allowing the majority to process the dissenting views extensively.

## Introduction

Groups are extensively used in higher education in order to promote the transfer and acquisition of curricular knowledge (Lou et al., 2001; Chen et al., 2004; Curşeu and Pluut, 2013; Kamau and Spong, 2015; de Hei et al., 2016). In collaborative learning, student groups engage collectively in educational activities in order to develop shared curricular knowledge (Lou et al., 2001; Van den Bossche et al., 2006; Nussbaum, 2008; Hämäläinen and Vähäsantanen, 2011). Beyond cognitive benefits derived from peer influences, collaborative learning groups also generate cognitive structures that surpass a simple aggregation of individual cognitions (Curşeu et al., 2007; Curşeu and Pluut, 2013). In other words, when students engage with curricular knowledge through working in groups, they can develop a collective understanding of the study material that transcends individual knowledge and comprehension. Group cognitive complexity (GCC) refers to the richness of these collective knowledge structures that is generated by the interplay of individual cognitive structures during interpersonal and intragroup interactions (Gruenfeld and Hollingshead, 1993; Curşeu et al., 2007).

Research to date has explored various antecedents of GCC ranging from individual cognitive complexity (Curşeu et al., 2007) to group diversity (Curşeu and Pluut, 2013) with the observation that the quality of interpersonal and intragroup interactions in collaborative learning groups is key in allowing integration of individual knowledge into collective emergent knowledge structures. The emergence of complex cognitive structures at the group level requires both cognitive differentiation and integration (Gruenfeld and Hollingshead, 1993). Through dialogue and argumentation, group members develop a better understanding of the curricular knowledge (Nussbaum, 2008; Nussbaum and Edwards, 2011). That is, group members have to engage in debates and task-related disagreements in order to generate the requisite variety for complex collective structures, yet they also need to converge in order for the collective structure to reach stability (Nussbaum and Schraw, 2007; Curşeu et al., 2012a).

Active minorities (group members that express views that differ from those held by the majority) have been shown to generate cognitive differentiation both for individual group members (Moscovici, 1980) as well as for the group as a whole (Nemeth, 1985, 1986; De Dreu and West, 2001). Minority dissent, however, is associated with relationship conflict and social rejection (Curşeu et al., 2012b) as dissenters often threaten the social harmony of the group. An open and accepting group climate could, however, generate the integrative potential needed for the effective integration of diverse points of view and thus render minority dissent effective in generating cognitive complexity. The aim of this paper is to test the interplay between minority dissent and social acceptance as antecedents of GCC.

### Minority Dissent and GCC

In general terms, dissent describes “the expression of disagreement with group norms, group action or a group decision” (Jetten and Hornsey, 2014, p. 463) and in particular, minority dissent describes situations in which one group member or a minority faction in a group expresses views that contradict the ones expressed by the majority of the group (Nemeth, 1986, 2012; Curşeu et al., 2012b). Moscovici’s minority dissent experiments showed that active minorities trigger cognitive conflict in the minds of the remaining group members and have the potential to lead to important (individual level) cognitive changes (Moscovici et al., 1969). Building on these insights on minority influence (Moscovici, 1976), subsequent research showed that minority dissent has important group level consequences as well (Nemeth, 1986; De Dreu and West, 2001; Curşeu et al., 2012b). In particular, minority dissent fosters group creativity and decision quality (Nemeth, 1985, 1986, 2012), innovation (De Dreu and West, 2001) and stimulates knowledge elaboration in groups (Curşeu et al., 2012b). In her extensive work on minority influence, Nemeth argues that minority dissent stimulates reflective processes in groups and divergent thought, which in turn are reflected in better information processing. A more recent paper (Curşeu et al., 2012b) supports these claims and shows that indeed minority dissent has a positive influence on GCC. Minority dissent is conducive for innovation and creativity in groups (Nemeth, 1985; De Dreu and West, 2001) and it increases the depth of information processing (Van Dyne and Saavedra, 1996; Nussbaum, 2008; Nussbaum and Edwards, 2011) and decision quality in groups (Schulz-Hardt et al., 2006). In line with the positive influence of minority dissent on the depth of information processing in groups we hypothesize that:

Hypothesis 1: *Minority dissent has a positive influence on group cognitive complexity.*

### Social Acceptance and GCC

Building on a social exchange perspective, the idiosyncrasy credits (IC) model (Hollander, 1958) argues that the group awards “interpersonal credits” to its individual members for behaviors that are aligned with normative expectations. In other words, socially desirable behaviors that preserve the social harmony within the group increase one’s interpersonal credit, while dissent as a disruptive interpersonal behavior is taxing on one’s interpersonal credit in the group. A group member can be influential only to the extent to which its interpersonal IC does not run out (IC > 0). A corollary of the IC model (Hollander, 1958) for minority influence is that cognitive change induced by minority dissent is most effective when the dissenter first conforms to the group (accumulates interpersonal credit) and only then engages in dissent. Bray et al. (1982) tested this claim against Moscovici’s approach to minority influence (Moscovici, 1980) and showed that indeed minority dissent was most effective when it followed initial conformity with majority opinions. According to Hollander (1958) an accepting group climate is an important factor for the acquisition of IC. Members in groups that display an accepting climate have a higher IC and thus more legitimacy in engaging in dissent as compared to members of groups in which social acceptance is low. As a consequence we expect that an accepting group climate increases the interpersonal IC of its members and, as such, minority dissent (MD) will be more effective than in groups that lack an accepting climate.

As argued, overt dissent often threatens the social harmony in groups and minority dissent triggers social rejection and relationship conflict (Curşeu et al., 2012b). In other words, dissent seems to have both positive and detrimental influences on information processing in groups. On the one hand, then, minority dissent is beneficial for the emergence of group cognition because it stimulates the depth of information processing (reflective and divergent thought processes) and, on the other hand, is detrimental for group atmosphere as it generates a negative affective climate (social rejection and relationship conflict). The dissenter leaving the group has (ironically) positive effects on GCC due to the diffusion of threat associated with minority dissent (Curşeu et al., 2012b). In other words, groups that can mitigate the threat associated with minority dissent seem to reap the cognitive benefits of minority dissent.

Previous research regarding the emergence of GCC shows that unequal participation is detrimental for GCC (Curşeu et al., 2010b) and the quality of interpersonal interactions has a positive effect on GCC (Curşeu et al., 2010a). In other words, GCC needs both cognitive differentiation (minority dissent, diversity of points of view) as well as social integration (high quality interpersonal interactions, good teamwork quality). In line with these arguments we argue that if the dissenter is accepted and the dissenting behavior is perceived by the group members as being helpful, the cognitive benefits of dissent can be enhanced. The divergent views expressed by a minority are most likely to increase GCC when the group climate is open to divergent contributions (e.g., a socially accepting group climate). We therefore hypothesize that:

Hypothesis 2: *Social acceptance increases the positive association between minority dissent and GCC.*

### Group Size, Minority Dissent, and GCC

In collaborative learning research, group size is one of the most common control variables as it can influence a variety of group (learning) processes and outcomes. On the one hand, group size is expected to be beneficial for group learning effectiveness as it is indicative of the knowledge pool and expertise members bring to the group, with larger groups benefiting from a more consistent knowledge base and expertise (Horowitz and Bordens, 2002) as well as from better error detection and correction (Hinsz, 2015). On the other hand, group size has a negative association with group learning effectiveness as larger groups tend to experience more process losses (social loafing, social inhibitions, diffusion of responsibility, Latané and Nida, 1981), more affective conflict (Amason and Sapienza, 1997) and less social support and more relational losses (Mueller, 2012).

Previous research on GCC reports very small correlations between group size and GCC (*r* = 0.08 in Curşeu et al., 2010b; *r* = -0.02 in Curşeu and Pluut, 2013, *r* = -0.06 in McNamara et al., 2002). These results are in line with meta-analytic evidence showing that group size has no linear association with collaborative learning effectiveness in groups (ρ = -0.02, see Tomcho and Foels, 2012). It is plausible that these small correlations can be explained by an underlying non-linear association between group size and GCC. This non-linear association reflects the co-existence of two mechanisms (one cognitive and one social-relational) that explain the association between the group size and GCC. The cognitive mechanism refers to the diversified, non-redundant knowledge that is entered in group debates by each additional group member and it explains the positive association between group size and GCC. The socio-relational mechanisms refer to coordination costs, social loafing, decrease of helping behaviors and social support as group size increases and it explains the negative association between group size and GCC.

The co-existence of these two mechanisms is also supported by meta-analytic evidence (Lou et al., 1996) showing that collaborative learning is effective in dyads (*d* = 0.15) or in groups of 3–4 (*d* = 0.22), and it is not effective when the group size varies between five and seven members (*d* = -0.02). The cognitive mechanism is supported by another meta-analysis that integrated the results of studies that compared knowledge transfer in individual learning compared with learning in a dyad or triad that reported a positive effect of collaborative learning on knowledge transfer in these small group settings (Pai et al., 2015).

Previous research (Hoegl, 2006; Mueller, 2012) already suggested that depending on the group task, an optimal group size could be identified in relation to collective performance. We therefore use a benefits-costs framework to argue that the initial cognitive benefits experienced as group size increases from 3 to 5 or 6 members are overcompensated for by the relational hindrances when the group size further increases to seven or eight members. We therefore hypothesize the following:

Hypothesis 3: *Group size has a non-linear association with GCC, in such a way that for low to average group size, the relationship between group size and GCC is positive, while for average to high group sizes, the positive relationship diminishes.*

## Materials and Methods

### Sample and Procedure

Five hundred and thirty seven students (256 women, *M*_AGE_ = 23.35), organized in 92 groups, each having 3–8 members participated in the study. All students were enrolled in a Dutch university and were engaged in collaborative learning as part of their curricular activities. During the semester, the groups had to solve case studies and perform different group exercises. As part of the course evaluation, each group was asked at the end of the semester to generate a cognitive map that captured their group understanding of the conceptual domain of the course (the course included topics like organizational diversity, conflict, team design and effectiveness, virtual organizations, multi-team systems). Each group was then asked to rate its cognitive map by comparing it to a map generated by two experts in the field. The self-rated grade covered 15% of the final course grade. After the cognitive mapping session at the end of the course, group members were asked to fill out a questionnaire and evaluate their way of working together during the cognitive mapping session.

### Ethics Statement

The data collection for the current study started in 2010 and according to the Dutch national ethical guidelines at the onset of the project, studies based on questionnaires that do not require any personal data with the potential to embarrass the participants were exempt from ethical committee approval. As the study was carried out as part of course related activities and no foreseeable risks beyond those present in regular curricular activities were anticipated, we did not ask for further approval from the local IRB.

### Measures

*Social acceptance* was evaluated with three items (“My ideas were fully accepted by the other team members,” “My team members appreciated my contributions to the group debates,” “I was treated as a marginal person by the other team members and my contributions were often disregarded” – reversed coded). Cronbach’s alpha for this scale is 0.758. Because the group level scores will be used for further analyses, we used the aggregation statistics to support the aggregation of individual scores to the group level. Within-group agreement index (Rwg, James et al., 1993) and the intraclass correlation coefficients (ICCs, Bliese and Halverson, 1998) are two of the most frequently used aggregation statistics in small group research (Woehr et al., 2015). The within-group agreement index is based on the within-group standard deviation of the individual scores while for computing the ICCs we used Bliese and Halverson’s (1998) formulas, based on the one way random effects analysis of variance:

$$\begin{array}{c} \mathrm{ICC}(1)=\frac{MS_{b}\;-MS_{w}}{MS_{b}\;+((N_{g}\;-1)\times \;MS_{w})}, \end{array}$$

where: MS_b_ is mean square between subjects, MS_w_ is mean square within subjects and N_g_ is the arithmetic mean of group sizes. Values of the ICC(1) refer to the amount of variance in a variable that can be attributed to the group and also reflects the extent to which individual scores within groups can be used to estimate the aggregated variable (Woehr et al., 2015). The ICC(2) is an indicator of group-mean reliability and it is computed based on the ICC(1) values using the following formula:

$$\begin{array}{c} \mathrm{ICC}(2)=\frac{N_{g}\;\times \;(\mathrm{ICC}(1))}{1+(N_{g}\;-1)\times \;\mathrm{ICC}(1)}. \end{array}$$

For our sample, the within-group agreement index ranges from 0.70 to 1.00 with an average of 0.91. The ICC(1) is 0.23 and ICC(2) is 0.63. In line with the guidelines presented in Woehr et al. (2015), based on these values of the aggregation statistics, we can conclude that the individual scores can be aggregated at the group level.

*Minority dissent* was evaluated with four items presented in Curşeu and ten Brink (2016) (e.g., “One or two team members often expressed ideas completely different than those of the other team members”). Cronbach’s alpha for this scale is 0.848. Although the individual group members are the unit of observation, minority dissent reflects the groups as entities. Therefore, before aggregating the individual scores to the group level, we computed several aggregation statistics. The within-group agreement index ranges from 0.74 to 1.00, with a mean of 0.91. The ICC(1) is 0.36 and the ICC(2) is 0.77. Therefore, aggregation at the group level is appropriate.

*Group cognitive complexity* was evaluated using a group cognitive mapping procedure described in Curşeu et al. (2007). Groups were given twenty key concepts extracted from the course material and were asked to organize these concepts on an A3 page in a way that reflected their collective understanding of the concepts and their relationships. After arranging the concepts, the students were instructed to draw and label the connections between them. Three cognitive map indicators were evaluated by an external rater, namely, map connectivity (the number of links between concepts), map diversity (the number of different types of connections established between concepts: causal, associative, structural, equivalent, topographical, hierarchical) and the number of concepts used in the map. The cognitive complexity of the map was computed using the relative complexity formula presented in Curşeu et al. (2007): GCC = (connectivity^∗^diversity)/number of concepts. This index captures groups’ integrative cognitive complexity (Gruenfeld and Hollingshead, 1993) as it reflects both the degree of cognitive differentiation (number of concepts and the diversity of connections among them) as well as integration (the number of connections among the concepts).

*Percentage of women in the group* was used as a control variable to account for the positive effect of gender diversity on GCC (Curşeu et al., 2007; Curşeu and Pluut, 2013). Because groups varied in size and minority influence may vary as a function of group size, we also accounted for the variance in group size in the analyses.

## Results

Means, standard deviations and bivariate correlations are presented in **Table 1**.

**Table 1:** Mean, standard deviation, and correlation.

	Mean	*SD*	1	2	3	4	5
(1) Group size	5.85	0.97	1				
(2) Percentage women	47.91	30.46	–0.06	1			
(3) Mean age	23.35	1.10	0.11	–0.28^∗∗^	1		
(4) Minority dissent	2.75	0.55	–0.02	–0.28^∗∗^	0.18	1	
(5) Social acceptance	4.10	0.33	–0.07	0.03	–0.07	–0.29^∗∗^	1
(6) GCC	3.89	1.63	0.18	0.24^∗^	–0.00	0.14	0.17

*^∗^*p* < 0.05, ^∗∗^*p* < 0.01.*

In order to test the hypotheses, we ran an OLS stepwise regression. In the first step we entered the group size, percentage of women, minority dissent and social acceptance (Model 1 in **Table 2**), in the second step the cross-product term of minority dissent and social acceptance (Model 2 in **Table 2**), and in the third step the cross product of group size and minority dissent as well as the quadratic term for group size (Model 3 in **Table 2**). In line with social impact theory, as the number of majority members increases so does their willingness to resist minority influence (Latané and Wolf, 1981; Wood et al., 1994). Therefore, in order to control for the extent to which the relational hindrances associated with large group size influence the impact of minority dissent we also entered the cross-product term between group size and minority dissent in the third step.

**Table 2:** Results of the OLS regression analyses for group cognitive complexity (*N* = 92).

	*Model 1*		*Model 2*		*Model 3*		*Model 4*	
	B (*SE*)	95%BCCI	B (*SE*)	95%BCCI	B (*SE*)	95%BCCI	B (*SE*)	95%BCCI
***Main effects^a^***								
Group size (GS)	0.39 (0.16)^∗^	[0.07; 0.71]	0.43 (0.16)^∗∗^	[0.12; 0.75]	0.37 (0.16)^∗^	[0.05; 0.69]	0.31 (0.17)^†^	[-0.03; 0.64]
Percentage of women	0.02 (0.01)^∗∗^	[0.01; 0.03]	0.02 (0.01)^∗∗^	[0.01; 0.03]	0.02 (0.01)^∗∗^	[0.01; 0.03]		
Minority dissent (MD)	0.95 (0.31)^∗∗^	[0.34; 1.57]	0.87 (0.31)^∗∗^	[0.27; 1.48]	0.81 (0.31)^∗∗^	[0.21; 1.42]	0.50 (0.31)	[-0.11; 1.11]
Social acceptance (SA)	1.34 (0.50)^∗∗^	[0.34; 2.34]	1.08 (0.50)^∗^	[0.09; 2.08]	1.13 (0.51)^∗^	[0.12; 2.14]	0.99 (0.52)^†^	[-0.04; 2.03]
**Interaction effects**								
MD × SA			1.92 (0.82)^∗^	[0.29; 3.55]	2.16 (0.82)^∗^	[0.53; 3.80]	1.98 (0.86)^∗^	[0.27; 3.68]
MD × GS					0.15 (0.40)	[-0.65; 0.95]		
Group size squared					-0.23 (0.11)^∗^	[-0.44; -0.02]	-0.25 (0.11)^∗^	[-0.46; -0.03]
***Conditional effects MD***								
Low SA			0.25 (0.43)	[-0.60; 1.10]			-0.15 (0.44)	[-1.03; 0.74]
Average SA			0.87 (0.30)^∗∗^	[0.27; 1.48]			0.50 (31)	[-0.11; 1.11]
High SA			1.50 (0.38)^∗∗^	[0.74; 2.26]			1.14 (0.38)^∗∗^	[0.37; 1.90]
R_sq_	0.21		0.26		0.30		0.20		
*F* change	5.81^∗∗^		5.46^∗^		2.40^†^		4.21^∗∗^		

*^a †^*p* < 0.10, ^∗^*p* < 0.05; ^∗∗^*p* < 0.01, unstandardized regression coefficients are presented in the table, SE, standard error, BCCI, bias corrected confidence intervals.*

**FIGURE 1:**
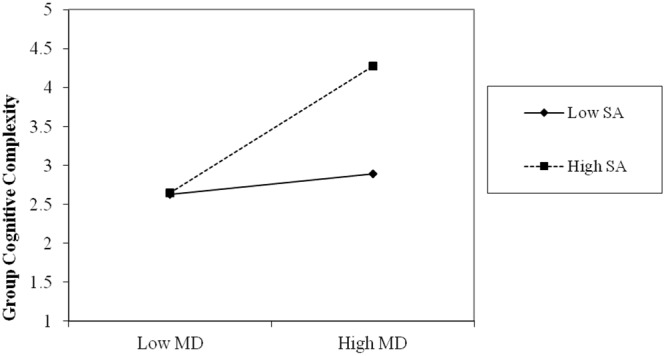
**The interaction effect of minority dissent and social acceptance on group cognitive complexity (GCC).** MD, minority dissent; SA, social acceptance.

Following the procedure described in Aiken et al. (1991), group size, minority dissent and social acceptance were centered before entering their cross-product term in the regression, in order to reduce multicollinearity. The results of the stepwise regression are presented in **Table 2**. As predicted, the effect of minority dissent was positive and significant (β = 0.32, *p* = 0.003), therefore the first hypothesis is supported. Moreover, the addition of the cross-product term added significantly to the predictive power of the model *F*(1,86) = 5.39 (*p* = 0.02), and the effect was significant (β = 0.22, *p* = 0.02). Therefore, we can conclude that Hypothesis 2 was supported too. We used the “pick-a-point” procedure described in Hayes and Matthes (2009) to estimate the conditional effect of minority dissent on GCC (see **Table 2** for the results). This method allowed us to estimate the effect of minority dissent (our focal predictor) at low (one standard deviation below the mean), average (sample mean) and high (one standard deviation above the mean) representative values of social acceptance (our moderator variable). We have used the SPSS macro described in Hayes and Matthes (2009) to compute the conditional effects of minority dissent on GCC. The conditional effects of minority dissent on GCC depending on social acceptance show that at low levels of social acceptance the effect of minority dissent on GCC was not significant, while at average and high levels of social acceptance the effect of minority dissent on GCC was positive and significant. **Figure 1** depicts the interaction effect of minority dissent and social acceptance on GCC.

**FIGURE 2:**
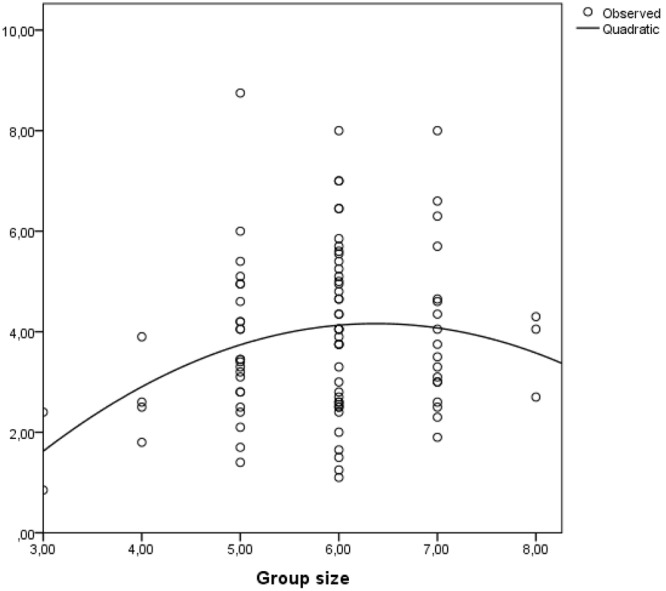
**The non-linear association between group size and GCC**.

The percentage of women had a positive association with GCC (β = 0.33, *p* = 0.001), meaning that groups with more women developed a more complex collective cognition than groups with fewer women. This outcome is in line with the results presented in Woolley et al. (2010), showing that the percentage of women in groups is a positive predictor of collective intelligence. Moreover, group size had a positive association with GCC (β = 0.22, *p* = 0.02), meaning that larger groups developed more complex representations than small groups. However, the quadratic term of group size had a significant negative association with GCC (β = -0.22, *p* = 0.025), that next to the significant positive effect of group size support the non-linear association between group size and GCC. We therefore conclude that Hypothesis 3 is supported. The non-linear association between group size and GCC is depicted in **Figure 2**.

The interaction effect of group size and minority dissent was not significant (β = 0.04, *ns*), therefore in our sample, the influence of minority dissent on GCC is not moderated by group size. However, in the whole sample, six groups were composed of four members or less, and because our measure of minority dissent referred to one or two members as a minority, we repeated our analyses excluding these six groups from the sample and the results did not change. Following one of reviewer’s comments, in order to further check the robustness of our results we ran the OLS analyses without the control variables (gender diversity and the interaction term between group size and minority dissent). The results are presented in Model 4 (**Table 2**). The interaction effect between MD and SA is significant and the quadratic effect of group size also remained significant, therefore we conclude that our results concerning group size and minority dissent are robust.

## Discussion

Our study provides further evidence for the positive association between minority dissent and knowledge elaboration in groups. Moreover, our study shows that social acceptance has a positive association with GCC, and, as such, we provide empirical evidence for the fact that complex emergent group cognition needs social integration. As argued by Gruenfeld and Hollingshead (1993) GCC requires both differentiation and integration. In order to develop cognitively complex representations, groups need to explore a wide variety of facts and task-related knowledge (differentiation) and to be able to establish numerous links among these constructs included in the representations (integration). Minority dissent is most certainly a driving force for differentiation, as divergent views expressed by minorities trigger cognitive conflict and stimulate divergent thinking (Nemeth, 1985, 1986; Nemeth et al., 2001). Social acceptance eventually stimulates the knowledge integration and thus increase the integrative potential of the group. These results are aligned with the positive indirect effects of social network differentiation (number of cliques or network fragmentation) and integration (network density) on GCC (Curşeu et al., 2012a). Future research could explore other factors that may stimulate knowledge differentiation and integration in groups as antecedents for GCC.

Group size has a non-linear association with GCC, with an inflection point at group sizes of five or six members. In other words, the positive association between group size and GCC reaches a plateau for group sizes of five or six members and then it turns negative. This finding has important implications for deciding on the optimal group size in collaborative learning settings in order to balance the cognitive benefits of having more resources with the detriments of social loafing and coordination costs. Further research could use the antecedents-benefit-costs framework (Busse et al., 2016) to further explore the two mechanisms (cognitive and socio-relational) that could explain the non-linear association between group size and GCC.

An emergent result refers to the positive effect of the percentage of women in groups on GCC. This result is similar to empirical evidence from the collective intelligence research (Woolley et al., 2010), showing that the percentage of women is positively associated to group intelligence (groups’ capacity of effectively solving a variety of cognitive tasks). It is argued that due to their higher social sensitivity, women create and preserve good interpersonal relations in groups and ultimately increase collective performance (Woolley et al., 2010). Moreover, the percentage of women is positively associated to the collective emotional intelligence of groups (Curşeu et al., 2015) and ultimately to group performance. Therefore, the plausible mechanism that explains the positive association between the percentage of women in groups and GCC is teamwork quality. Future research could further explore this claim.

Because teamwork skills are essential for employability in modern organizations (Chen et al., 2004) universities adapted their education programs to include group work (Riebe et al., 2010) in which students can exercise and develop their teamwork skills. Acquiring teamwork skills, however, is not a trivial endeavor as group work in higher education is often marked by conflict and tensions (Kamau and Spong, 2015). Although beneficial in many respects, minority dissent can have negative consequences and dealing with these consequences is a key component of teamwork skills. As our results show, building a group climate that is accepting toward individual contributions is one path that can be followed to deal with the negative consequences of minority dissent. In practical terms, our study shows the necessity of having both minority dissent as well as a socially accepting group climate.

### How to Generate Dissent and Social Acceptance in Groups?

As illustrated by our results, if groups develop a socially accepting climate, the cognitive benefits of dissent can be enhanced. Social acceptance of dissent can be induced through group norms or process interventions. Groups can be trained to use a number of “ground rules” while working together; ground rules that help create an accepting climate that also allows for dissent. The benefits of normative frameworks in the emergence of group cognition has previously been documented (Curşeu and Schruijer, 2012).

Another way of stimulating constructive cognitive conflict in groups and enhance the level of participation in collaborative discourse is the use of argumentation-vee-diagrams (Nussbaum, 2008; Nussbaum and Edwards, 2011). The graphic technique of argument-counterargument integration, allows students to follow a structured dialogue and actively engage in dissent. The argument-counterargument integration using the graphical support of diagrams (Nussbaum and Schraw, 2007) will ensure both differentiation (exploration of various aspects in a cognitive schema) as well as integration (in depth consideration of counter-arguments during the debate through refutation, synthesis and weighing) that are essential for the emergence of cognitively complex collective representations.

Decision techniques as the “devil’s advocacy” could also be used to create minority dissent, although research to date shows that natural dissent is more effective than contrived dissent in generating high quality decisions (Nemeth et al., 2001; Schulz-Hardt et al., 2002). Such a technique involves appointing a typical “devil’s advocate” role within the group and the person holding that role will eventually criticize the preferences shared by the majority. Role rotation can be used in order to reduce the social rejection and the negative consequences of repeatedly playing this role and in this way group members could develop a more accurate understanding of the intricacies of disagreeing with the group and ultimately be more accepting when this behavior is enacted by someone else.

Finally, elements of diversity awareness training could be adopted to create short interventions aimed at increasing awareness of the benefits of minority dissent. Diversity awareness training was originally introduced to alleviate some of the negative consequences of group diversity, mostly rooted in social categorization processes (Robertson et al., 2001). As minority dissent is actually a form of cognitively enacted diversity (a minority expresses opinions that contradict the status quo in the group), elements of diversity awareness training could help group members understand their natural tendency of distancing themselves from a dissenter and to grow more accepting of dissent in their group’s midst.

### Limitations

Next to its contributions this study has several limitations. First, none of the independent variables considered here were directly manipulated and the study is cross-sectional. Therefore, no causal claims can be inferred. Second, as the study is cross-sectional and the data regarding the independent variables were collected from the same source, common method bias is a concern (Podsakoff et al., 2011). However, in order to mitigate this concern, GCC was evaluated by an independent rater. Therefore, the dependent variable evaluation comes from a different source. Moreover, as illustrated by Evans (1985), common method bias is less of a concern for studies testing interactions as the interaction effect estimated in the linear regression is not likely to be an artifact of common method bias (Siemsen et al., 2010). Third, the instructions used in the cognitive mapping exercise (group members had to reach consensus on the cognitive map structure) could have enforced artificial consensus. In different contexts, the emergent group level cognitive structures could actually be more volatile and difficult to capture.

## Author Contributions

Conceived and designed the study: PC, SS, and OF. Collected the data: PC. Analyzed the data: PC and OF. Wrote the paper: PC, SS, and OF.

## Conflict of Interest Statement

The authors declare that the research was conducted in the absence of any commercial or financial relationships that could be construed as a potential conflict of interest.
